# Quantitative Comparison of Two Novel Swept-Source Optical Coherence Tomography Angiography Devices

**DOI:** 10.3390/diagnostics16050801

**Published:** 2026-03-08

**Authors:** Michael Hafner, Daniel J. P. Deschler, Alexander Kufner, Lisa M. Katscher, Siegfried G. Priglinger, Maximilian J. Gerhardt

**Affiliations:** Department of Ophthalmology, LMU University Hospital, Ludwig-Maximilians—Universität München, Mathildenstraße 8, 80336 Munich, Germany

**Keywords:** quantitative OCT angiography, swept-source OCTA, retinal capillary plexuses, microvascular metrics, device comparison, en face imaging

## Abstract

**Background**: Swept-source optical coherence tomography angiography (SS-OCTA) enables rapid assessment of retinal microvasculature. However, cross-platform comparability remains limited by device-specific acquisition and image quality characteristics. This study prospectively compared two novel SS-OCTA systems, DREAM (200 kHz) and BMizar (400 kHz). **Methods**: Fifty eyes from 25 healthy participants underwent 3 mm × 3 mm macular OCTA imaging with both devices in a single session. Images were analysed using OCTAVA to extract foveal avascular zone (FAZ) area, vessel area density (VAD), total vessel length (TVL), node counts, fractal dimension (FD), median vessel length (MVL) in SCP, and mean vessel diameter (MVD) in DCP. Image quality was assessed using FAZ-noise rate, contrast-to-noise ratio (CNR), and FAZ noise-floor standard deviation. Paired comparisons were performed using Wilcoxon signed-rank tests and Cliff’s delta. **Results**: BMizar acquisition time was shorter than DREAM for the evaluated 3 × 3 mm protocol (median 5.36 s vs. 9.93 s), reflecting differences in A-scan rate and protocol implementation; acquisition time is therefore reported descriptively. In the SCP, DREAM yielded lower VAD (41.9% vs. 48.8%) and fewer nodes (1547 vs. 1879) but exhibited markedly less background noise (noise-floor SD 4.1 vs. 57.9) and substantially higher CNR (16.7 vs. 0.82). DREAM also showed longer MVL (45 vs. 39 µm) and higher FD (1.98 vs. 1.97; δ = 0.90). In the DCP, DREAM demonstrated smaller FAZ areas (0.27 vs. 0.42 mm^2^), thinner MVD (14 vs. 25 µm), higher node counts (3144 vs. 2301), longer TVL (223.6 vs. 206.2 mm), and higher FD (1.98 vs. 1.97), whereas VAD was higher on BMizar (32.96% for DREAM vs. 49.93% for BMizar). FAZ-noise rates were consistently higher for BMizar in both plexuses. **Conclusions**: Both devices provide reliable SS-OCTA imaging, but with distinct strengths. DREAM delivers higher vascular continuity and more reliable FAZ and DCP quantification, whereas BMizar achieves faster acquisition at the cost of noise, inflating SCP density and distorting FAZ-based metrics. Awareness of these characteristics is essential to ensure the valid use of OCTA biomarkers in clinical and research applications.

## 1. Introduction

Since its clinical adoption, optical coherence tomography angiography (OCTA) has become a key modality for assessing chorioretinal vascular pathology [1]. In contrast to dye-based angiography (fluorescein/ICGA), OCTA is performed without intravenous contrast administration, thereby avoiding injection-related adverse events and improving patient comfort [2]. OCTA permits fast, repeatable scans with high capillary resolution unaffected by dye leakage and depth-resolved imaging, enabling analysis of blood flow in specific retinal and choroidal layers [3]. It operates by detecting motion contrast from moving blood cells in sequential OCT B-scans, generating en face angiograms of the perfused vasculature [4].

Recent technological advances in swept-source OCTA (SS-OCTA) increased scanning speeds and imaging ranges. Intalight Inc. (San Jose, CA, USA) introduced the DREAM SS-OCTA system, initially approved in China, and subsequently in Brazil [5] and in Europe [6]. Providing a scanning frequency of 200 kHz, DREAM OCT significantly exceeds speeds of earlier spectral-domain and SS-OCTA devices. Similarly, TowardPi Medical (Beijing, China) recently developed the BMizar SS-OCTA (model BM-400K), which has a 400 kHz scan rate. The BMizar system received CE certification in 2024 [7]. (For context: the Heidelberg Spectralis scan rate is about 125 kHz [8]; Topcon Triton about 100 kHz [9]; and Zeiss Cirrus 68 kHz [10].)

High-speed SS-OCTA systems primarily aim to reduce acquisition time to improve patient comfort and workflow and to mitigate motion artefacts, which is particularly relevant when scan density and/or field-of-view are increased. However, acquisition duration, sampling density, the number of repeated B-scans, and motion-correction/averaging strategies are tightly coupled and can influence angiographic contrast, background noise, and the stability of downstream quantitative measurements. Prior work has shown that OCTA-derived quantitative endpoints are sensitive to image quality and noise characteristics, and that denoising/averaging can materially alter density- and topology-related metrics, emphasizing the need for standardized head-to-head evaluations when OCTA is used for biomarker-driven research or longitudinal monitoring [11,12,13].

With the emergence of additional high-speed SS-OCTA platforms, it becomes increasingly important to understand how device-specific acquisition and processing characteristics translate into image quality, noise profiles, and quantitative biomarker outputs under standardized conditions. Prior comparative work across commercial OCTA devices has demonstrated that qualitative appearance and quantitative metrics can differ substantially between platforms, even when nominal scan sizes are similar, highlighting the risk of pooling or comparing quantitative endpoints across systems without harmonization [14,15,16,17]. Therefore, direct head-to-head comparisons using identical scan settings and a unified analysis pipeline are clinically relevant, particularly for endpoints that are known to be noise- and quality-sensitive (e.g., DCP- and FAZ-derived metrics) [13,18].

In this prospective within-subject study, we performed a direct comparison of DREAM and BMizar under identical macular scan settings and quantified OCTA outcomes using the same non-proprietary analysis pipeline (OCTAVA) across both datasets. The aims were defined a priori as follows: (i) Primary aim: Quantify and compare image-quality/noise characteristics (including contrast-to-noise- and FAZ-noise-related robustness measures) and acquisition duration for the exact protocol tested. (ii) Secondary aim: Quantify and compare clinically relevant microvascular metrics in SCP and DCP (density/perfusion, topology/complexity, and FAZ-related endpoints) using a unified analysis workflow. (iii) Exploratory aim: Assess whether noise-related differences may induce systematic bias (e.g., inflation of density/branching metrics) and derive practical recommendations for clinical and research use.

## 2. Materials and Methods

The study protocol was approved by the Institutional Review Board of the Faculty of Medicine, LMU Munich (ID: 24-0571 and 25-0912), and was conducted in accordance with the Declaration of Helsinki. All participants provided written informed consent. Age and sex were recorded for all participants.

### 2.1. Imaging

Two SS-OCTA devices were used for imaging: the Intalight DREAM OCT (VG200D, software version v1.0.403A, Intalight Inc., USA) and the TowardPi BMizar 400 kHz OCT (software version VS15, TowardPi Medical, China). The DREAM OCT operates at a centre wavelength range of 1030–1070 nm, an A-scan rate of 200 kHz and an axial resolution of ≤5.5 µm as well as a transverse resolution of ≤15 µm [19]. The BMizar uses a laser centred at 1060 nm with a 400 kHz rate [20] and has an axial resolution of ≤6 µm and a transverse resolution of 10 µm. The DREAM system can achieve a maximum image depth of 12 mm in tissue, whereas the BMizar can reach an imaging depth of up to 6 mm in tissue [19,20].

**Figure 1:**
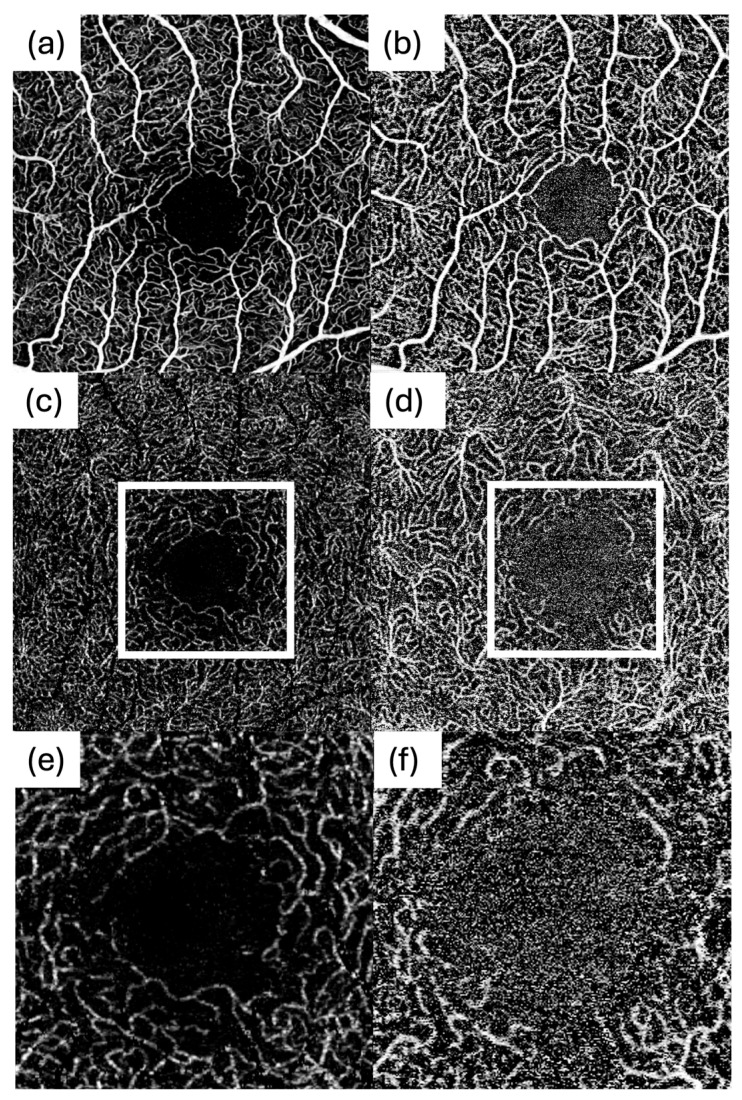
Representative 3 × 3 mm en face OCTA images from the same participant acquired with DREAM and BMizar. (**a**) SCP, DREAM; (**b**) SCP, BMizar. (**c**) DCP, DREAM; (**d**) DCP, BMizar. Magnified DCP views centered on the FAZ are shown for (**e**) DREAM and (**f**) BMizar.

For both systems, macular OCTA volumes covering a 3 mm × 3 mm field centred on the fovea were acquired. To maximize image quality, each system’s highest available lateral sampling density was used, which was 512 × 512 pixels for each device. All scans on both devices were set to acquire four repeated B-scans per position (Automatic Real Time setting of 4) to average out noise. Each device’s proprietary motion correction and eye tracking were active during acquisition. Each eye on both devices was imaged under similar conditions (same visit, with minimal time between scans to avoid physiological changes). Representative 3 × 3 mm en face OCTA images from the same participant acquired with DREAM and BMizar, including SCP and DCP slabs as well as magnified FAZ-centered views, are shown in Figure 1. For all eyes, imaging time was measured during OCTA acquisition.

### 2.2. Participants

Both eyes were eligible provided there was no history of chorioretinal disease and no prior ocular surgery that might influence retinal microvascular readouts. Participants were excluded if they reported systemic conditions known to affect the retinal microvasculature, including diabetes mellitus, arterial hypertension, or chronic kidney disease. This restriction to young, healthy eyes was intentional to provide high-signal, low-attenuation reference data and to isolate device- and acquisition-related differences under standardized conditions. Consequently, the present results may not fully translate to eyes with retinal pathology, in which signal attenuation (e.g., due to edema, hemorrhage, ischemia-related flow deficits, or media opacities) can increase noise and reduce segmentation robustness.

### 2.3. Image Analysis

Retinal OCTA scans were analysed with the OCTA Vascular Analyser (OCTAVA), an open-source software tool by Untracht et al. [21]. OCTAVA is designed for cross-platform OCTA image analysis and is operated as a MATLAB-based application. It was used on MATLAB R2023a for Windows (version 9.14.0.2306882, 64-bit, win64; MathWorks, Natick, MA, USA).

**Figure 2:**
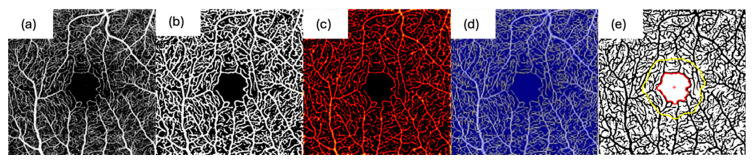
OCTAVA processing workflow illustrated on a DREAM OCTA en face image (512 × 512 px). (**a**) Input en face image. (**b**) Vessel enhancement and binarization after Frangi filtering. (**c**) Vessel diameter map derived from the Euclidean distance transform. (**d**) Skeleton representation after MATLAB 3D thinning. (**e**) Example of automated FAZ segmentation (red).

A two-dimensional Frangi filter (threshold value: three) was applied to enhance vessel visibility in the en face projections. Following this, vessel segmentation was carried out using fuzzy thresholding with an adaptive threshold kernel size of 70, separating pixels into vessel and non-vessel regions. Figure 2b illustrates the result after Frangi filtering and binarization.

The binarized images were then skeletonized using a 3D thinning algorithm in MATLAB, and vessel diameters were quantified by applying a Euclidean distance transform to generate a heatmap. To assess vascular network topology, the skeletonized image was converted into a graph. After branch point identification, based on connectivity, vessel segments were classified. Very short lines (shorter than the chosen twig size) and isolated structures were excluded as noise. Based on visual inspection, a twig size threshold of two pixels was applied. Figure 2c shows an example of a vessel diameter heatmap, and Figure 2d depicts a skeletonized en face image.

Based on the binarized images, the FAZ was segmented automatically, and the area was measured. The example for an automatically detected FAZ can be found in Figure 2e. All FAZ segmentations were visually verified and corrected if necessary. In some scans, high-intensity background noise contaminated the avascular zone and prevented reliable automatic FAZ initialization. In these cases, the FAZ was manually initialized (“seeded”) by placing an approximate initialization contour within the FAZ region, after which the identical OCTAVA FAZ segmentation workflow was executed. Importantly, this manual step served only as an initialization to guide automated segmentation and was not a freehand outlining of the final FAZ border.

Quantitative OCTA biomarkers were extracted with OCTAVA for both the superficial (SCP) and deep capillary plexus (DCP). To align measurements with clinical utility, endpoints were grouped into: (i) image-quality/noise robustness measures (contrast-to-noise ratio, FAZ-noise rate, and noise-floor variability) to quantify whether background noise and reduced angiographic contrast may compromise reliable quantification; (ii) perfusion/density measures (vessel area density), commonly used for ischemia assessment and longitudinal monitoring; (iii) topology/complexity measures (total vessel length, vessel length density, branching density, number of nodes, and fractal dimension), which are sensitive to spurious vessel detection in noisy data and therefore informative regarding robustness of quantitative readouts; (iv) FAZ metrics (FAZ area and FAZ-related robustness), widely used ischemia biomarkers that are particularly susceptible to boundary instability and noise contamination [12,13,22]. To further characterize vessel geometry beyond density and topology, median vessel length (MVL) was evaluated for the SCP and mean vessel diameter (MVD) for the DCP. All metrics were extracted using identical OCTAVA settings for both devices.

Further image analysis was performed using Fiji (ImageJ2, version 2.16.0/1.54p) [23]. For each image, three noise- and background-related quality metrics were calculated separately for SCP and DCP. The largest possible ellipsoid within the FAZ was manually delineated and eroded by two pixels to avoid contamination from parafoveal capillaries. Based on the pixel intensities in this eroded FAZ ($I_{FAZ}\;=\;\left\{I_{p}|||p\in FAZ\right\}$), a noise threshold $T_{FAZ}$ was defined as the 97.5th percentile of the FAZ intensity distribution over all images and systems analysed:

$$T_{FAZ}=P_{97.5}(I_{FAZ})$$

The FAZ-noise rate was then calculated as the fraction of FAZ pixels with intensities above this threshold:

$$R_{FAZ}=\frac{\left|\left\{p\in FAZ|||I_{p}>T_{FAZ}\right\}\right|}{\left|FAZ\right|}$$

A higher $R_{FAZ}$ indicates stronger contamination of the avascular zone by spurious high-intensity signals.

**Figure 3:**
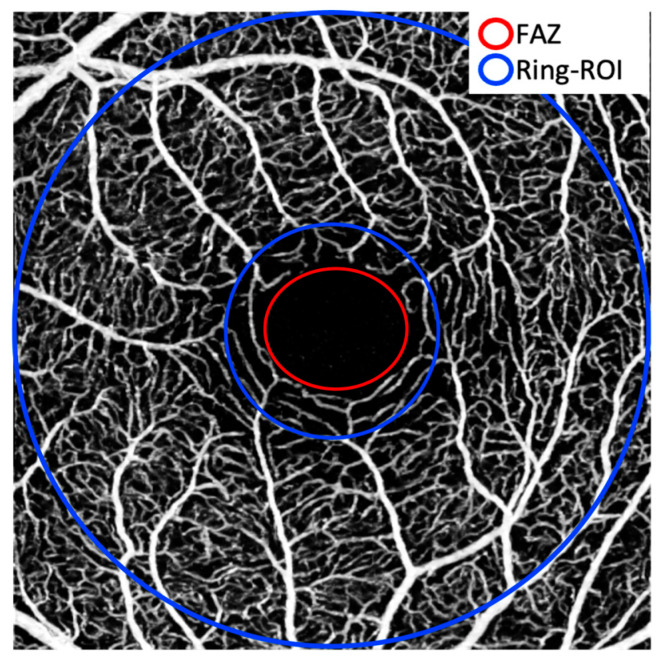
Visualisation of the FAZ delineation using the ellipsoid tool (red) and of the automatically generated ring-shaped ROI (blue).

In addition, the foveal centre was manually marked, and a ring-shaped region of interest (ROI) was defined as an annulus with inner radius 0.5 mm and outer radius 1.5 mm. Figure 3 presents an example of this procedure. The contrast-to-noise ratio (CNR) was calculated as:

$$CNR=\frac{\langle I_{Ring}\rangle -\langle I_{FAZ}\rangle }{{SD}_{FAZ}}$$

where $\langle I_{Ring}\rangle$ and $\langle I_{FAZ}\rangle$ denote the mean intensities within the ring ROI and FAZ, respectively, and ${SD}_{FAZ}$ is the standard deviation of FAZ pixel intensities.

Finally, the noise-floor standard deviation was defined directly as the standard deviation of FAZ pixel intensities:

$${SD}_{FAZ}=\sqrt{\frac{1}{\left|FAZ\right|-1}\sum_{p\in FAZ}{\left(I_{p}-\langle I_{FAZ}\rangle \right)}^{2}}$$

### 2.4. Data Analysis and Statistics

For each quantitative parameter, the values derived from DREAM and BMizar were compared. The distribution of each metric was checked for normality, applying the Shapiro–Wilk test. Given that many vascular metrics were not normally distributed, we planned to use non-parametric statistics. Paired comparisons were conducted using the Wilcoxon signed-rank test. Effect sizes for paired data were summarized using Cliff’s delta (δ), which reflects the probability that values from one device are systematically higher or lower than those from the comparator (δ ranges from −1 to +1; δ = 0 indicates no dominance). For completeness, δ can be expressed as δ=n+−n−n, where $n_{+}$ and $n_{-}$ denote the count of pairs in which DREAM’s value is larger or smaller than the BMizar value, respectively, and n is the total number of pairs. Acquisition duration was treated as a protocol-driven characteristic and is therefore reported as median (IQR) without inferential testing.

Calculations were done with Prism 10 for macOS (Version 10.5.0) and RStudio for Mac (2024.12.1+563). Statistical significance was set for *p* < 0.05. Because this study is a method-comparison/hypothesis-generating analysis and several OCTA metrics are correlated (e.g., density-, length-, branching-, and fractal-based measures), we did not apply a formal multiplicity correction. We therefore report unadjusted *p*-values as descriptive measures of evidence and interpret results primarily based on effect sizes and the consistency of direction across endpoints; isolated or borderline *p*-values should be interpreted with caution in the context of multiplicity and correlated outcomes.

## 3. Results

### 3.1. Baseline Demographics

For this study 50 eyes from 25 healthy participants were examined using the two different OCTA devices. No scans were excluded, ensuring that all acquired images contributed to the analysis. The average age of the patients was 24.96 ± 2.85 years (mean ± standard deviation), with 17 female and 8 male participants.

### 3.2. Acquisition Time

**Figure 4:**
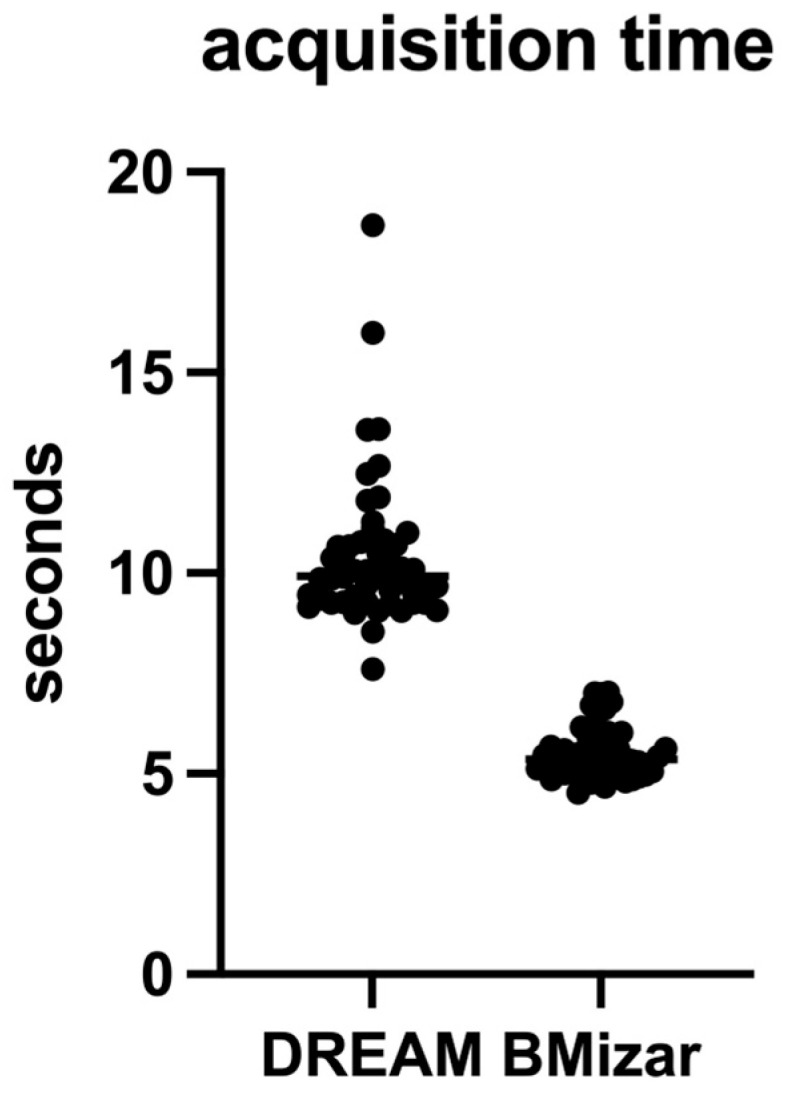
Acquisition duration for 3 mm × 3 mm OCTA scans obtained with the two imaging systems. Box plots display the median and interquartile range. Acquisition time is reported descriptively for the evaluated protocol.

For all 50 eyes, the duration of OCTA image acquisition was documented. The median acquisition time was 9.93 s (IQR: 1.503 s) when using the DREAM OCTA system, whereas a median time of 5.36 s (IQR: 0.835 s) was observed for the BMizar OCTA system. Acquisition time is reported descriptively because it is primarily determined by protocol settings and A-scan rate rather than an empirical hypothesis test. Detailed values are summarized in Table 1 and illustrated in Figure 4.

### 3.3. Superficial Capillary Plexus

Quantitative parameters of the superficial capillary plexus differed notably between the two OCTA systems. Vessel area density (VAD) exhibited a median value of 41.89% (IQR: 2.23%) when assessed with DREAM OCTA, whereas BMizar OCTA yielded a higher median of 48.83% (IQR: 1.63%). This difference was statistically significant (*p* < 0.0001; δ = −1.00). Analysis of the foveal avascular zone (FAZ) area revealed comparable measurements between devices. DREAM OCTA demonstrated a median FAZ size of 0.2565 mm^2^ (IQR: 0.1622 mm^2^), while BMizar OCTA measured a median of 0.2370 mm^2^ (IQR: 0.2060 mm^2^), with no statistically significant difference observed (*p* = 0.1413; δ = 0.14). Manual FAZ initialization (seeding) due to FAZ noise contamination was required in 0/50 DREAM scans (0%) and 3/50 BMizar scans (6%) for the SCP. Total vessel length (TVL) was significantly greater in images obtained with BMizar OCTA, showing a median of 188.1 mm (IQR: 7.7 mm), compared with 174.2 mm (IQR: 12.1 mm) for DREAM OCTA (*p* < 0.0001; δ = −0.84). Similarly, network complexity assessed by node count was higher for BMizar OCTA, which detected a median of 1879 nodes (IQR: 163), whereas DREAM OCTA identified in median 1547 nodes (IQR: 228) (*p* < 0.0001; δ = −0.92).

**Figure 5:**
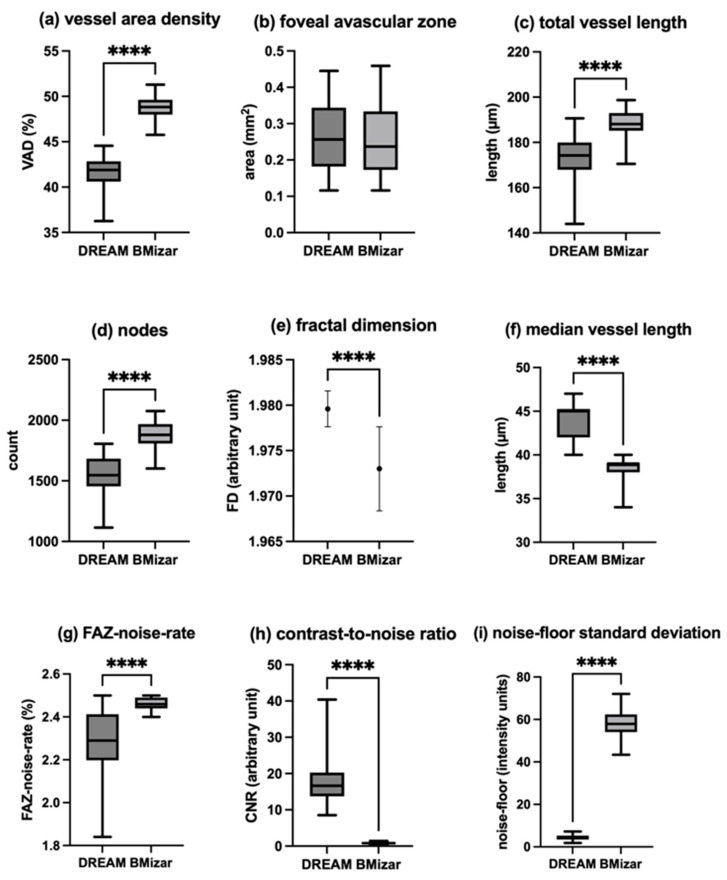
Comparison of superficial capillary plexus parameters obtained by the two OCTA systems. (**a**) Vessel area density. (**b**) Foveal avascular zone. (**c**) Total vessel length. (**d**) Number of nodes. (**e**) Fractal dimension. (**f**) Median vessel length. (**g**) FAZ-noise rate. (**h**) Contrast-to-noise ratio. (**i**) Noise-floor standard deviation. Statistically significant differences (*p* < 0.05) are indicated by asterisks (****, *p* < 0.0001).

Fractal Dimension (FD) analysis revealed a slightly higher mean value for DREAM OCTA (1.980 with a SD of 0.001979) compared with BMizar OCTA (1.973 with a SD of 0.004629), with this difference reaching statistical significance (*p* < 0.0001; δ = 0.90). In contrast, mean vessel length (MVL) was shorter in BMizar OCTA scans, with a median of 39 µm (IQR: 1.00 µm) compared to 45 µm (IQR: 3.25 µm) measured using DREAM OCTA (*p* < 0.0001; δ = 1.00). An overview of these quantitative vascular parameters is provided in Table 2 and illustrated in Figure 5. For variables not following a Gaussian distribution, variability is expressed using interquartile ranges.

For DREAM OCTA, a median FAZ-noise rate of 2.29% (IQR: 0.215%) and for BMizar a median of 2.44% (IQR: 0.050%) was measured (*p* < 0.0001; δ = −0.7). The CNR of Dream OCTA was determined as a median of 16.650 (IQR: 6.560) and 0.820 (IQR: 0.325) for BMizar OCTA (*p* < 0.0001; δ = 1). Finally, the calculated noise-floor-SD for DREAM OCTAs showed a median of 4.11 (IQR: 1.825) and a median of 57.90 (IQR: 8.330) for BMizar OCTAs (*p* < 0.0001; δ = −1.00). All relevant data can be found in Table 3 and Figure 5.

### 3.4. Deep Capillary Plexus

VAD was measured with 32.96% (IQR: 2.11%) for DREAM OCTA and 49.93% (IQR: 1.06%) for BMizar OCTA, with a statistically significant difference (*p* < 0.0001; δ = −1.00). Analysis of the FAZ showed a median area of 0.2665 mm^2^ for DREAM OCTA (IQR: 0.1613 mm^2^) and 0.4240 mm^2^ for BMizar OCTA (IQR: 0.1795 mm^2^) (*p* < 0.0001; δ = −0.92). For the DCP, manual FAZ initialization (seeding) was required in 1/50 DREAM scans (2%) and 10/50 BMizar scans (20%). TVL was higher for DREAM OCTA with a median of 223.6 mm (IQR: 14.6 mm) compared to 206.2 mm for BMizar OCTA (IQR: 6.9 mm) (*p* < 0.0001; δ = 0.84). Regarding the vascular network complexity, DREAM OCTA presented a median of 3144 nodes (IQR: 363), while BMizar OCTA identified 2301 nodes (IQR: 134) (*p* < 0.0001; δ = 0.96).

**Figure 6:**
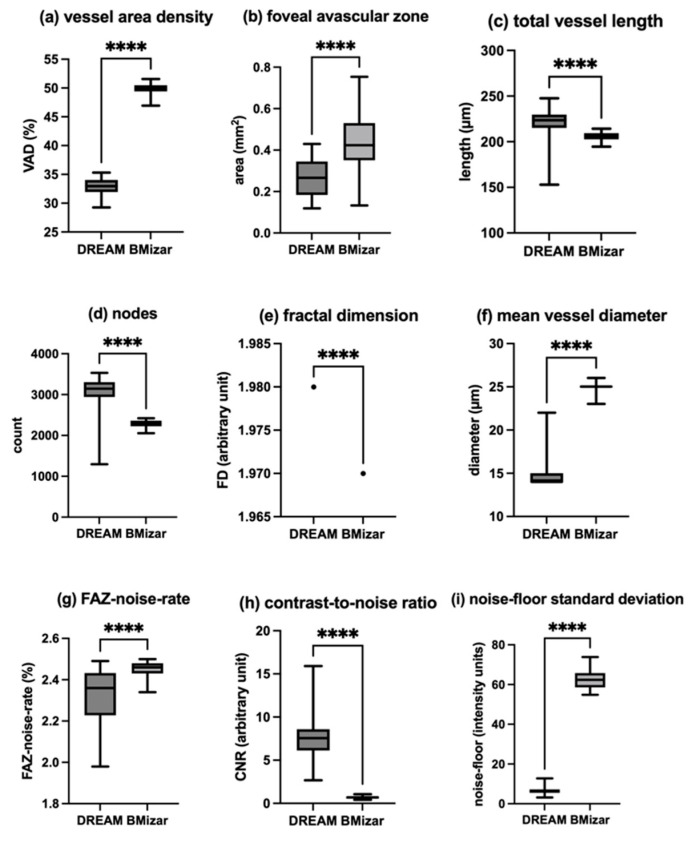
Comparison of deep capillary plexus parameters obtained by the two OCTA systems. (**a**) Vessel area density. (**b**) Foveal avascular zone. (**c**) Total vessel length. (**d**) Number of nodes. (**e**) Fractal dimension. (**f**) Mean vessel diameter. (**g**) FAZ-noise rate. (**h**) Contrast-to-noise ratio. (**i**) Noise-floor standard deviation. Statistically significant differences (*p* < 0.05) are indicated by asterisks (****, *p* < 0.0001).

FD analysis yielded mean values of 1.980 for DREAM OCTA and 1.970 for BMizar OCTA (both with very low variability; SD ≈ 0), with a statistically significant difference (*p* < 0.0001; δ = 1.00). MVD was determined at 14 µm (IQR: 1 µm) for DREAM OCTA and 25 µm (IQR: 1 µm) for BMizar OCTA (*p* < 0.0001; δ = −1.00). All values are summarized in Table 2 and illustrated in Figure 6.

For DREAM OCTA, a median FAZ-noise rate of 2.36% (IQR: 0.205%) and for BMizar OCTA a median of 2.46% (IQR: 0.050%) was measured (*p* < 0.0001; δ = −0.76). The CNR of Dream OCTA was determined as median of 7.560 (IQR: 2.462) and 0.685 (IQR: 0.235) for BMizar OCTA (*p* < 0.0001; δ = 1). Finally, the calculated noise-floor-SD for DREAM showed 6.15 (IQR: 1.760) and 62.36 (IQR: 7.190) for BMizar (*p* < 0.0001; δ = −1.00). All data can be found in Table 3 as well as Figure 6.

## 4. Discussion

To the best of our knowledge, this study presents the first direct quantitative comparison between the novel DREAM and BMizar SS-OCTA platforms. Both systems produced high-quality macular angiograms, yet systematic differences emerged. These differences were most prominent in acquisition speed and in quantitative SCP and DCP metrics, largely driven by image quality and noise characteristics. Our findings highlight important implications for both clinical decision-making and cross-platform research comparability.

**Figure 7:**
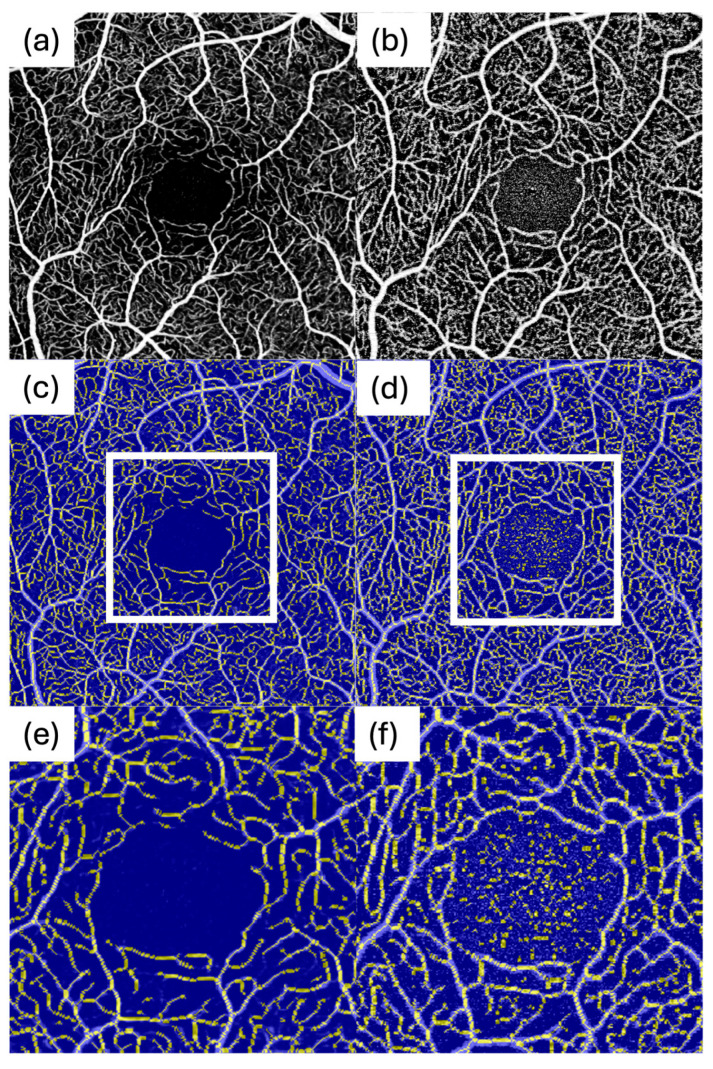
Comparison of OCTA image processing between DREAM and BMizar. The first row shows the original en face OCTA images for DREAM (**a**) and BMizar (**b**). The second row displays the corresponding images after binarization and skeletonization for DREAM (**c**) and BMizar (**d**). The third row presents magnified views of the foveal avascular zone (FAZ) from the binarized and skeletonized images in the second row for DREAM (**e**) and BMizar (**f**).

In the SCP, DREAM showed lower vessel area density and fewer nodes than BMizar. However, DREAM images exhibited substantially lower background noise and a much higher contrast-to-noise ratio. DREAM’s image quality resulted in smoother, longer vessel segments and fewer spurious signals inside the FAZ. BMizar’s higher noise rate was particularly evident in the FAZ (Figure 7b). Consistent with this, FAZ boundary detection was more frequently affected by noise on BMizar, necessitating manual FAZ initialization (seeding) in a subset of scans and underscoring that automated FAZ segmentation remains vulnerable to high-noise data. The higher background noise often led to misclassification of background speckle as vasculature, particularly in avascular areas (Figure 7f), artificially inflating SCP density and branching counts. Thus, while BMizar reported “denser” superficial networks, DREAM might more likely reflect the underlying anatomy. These findings echo earlier comparative studies that identified variability in SCP metrics across OCTA systems due to differences in acquisition speed and noise behaviour [14,15].

Interpretation of quantitative OCTA readouts across devices is challenging because metrics are sensitive to differences in acquisition settings, proprietary processing, and image quality. Comparative studies across commercial OCTA systems have reported notable inter-platform variability of quantitative endpoints, and work on OCTA image quality has shown that noise and artefacts can materially influence density- and topology-related measurements as well as FAZ stability [12,13,22]. These observations motivate standardized comparisons and support the use of unified, non-proprietary analysis pipelines when the goal is biomarker-driven interpretation rather than device-specific output.

In case of DCP, even more pronounced divergences were found. While in the SCP, FAZ areas were comparable between devices, DREAM consistently produced smaller FAZ areas and thinner mean vessel diameters in the DCP, together with higher node counts and longer total vessel length. These results indicate improved sensitivity for fine-caliber capillaries and more accurate delineation of the FAZ border. By contrast, BMizar yielded higher overall vessel density, a finding that, given the higher noise level, is likely to overestimate perfusion. Clinically, these differences are critical, as FAZ size and DCP morphology are well-established biomarkers for ischemia in vascular retinal diseases such as diabetic retinopathy [24].

FD, a global measure of vascular complexity, also differed between devices. In the SCP, DREAM exhibited higher FD values than BMizar. Although the absolute numerical difference was small, the large effect size indicates a systematic bias. DREAM’s cleaner background and improved vessel continuity likely enhanced the fractal representation of fine capillaries, increasing FD despite lower density and fewer nodes. BMizar’s elevated noise and fragmented skeletons, by contrast, appeared to lower FD in the SCP. A similar pattern showed in the DCP with DREAM again exhibiting slightly higher FD values than BMizar with a large effect size, supporting a consistent bias across plexuses.

Acquisition time should be interpreted as a protocol-driven characteristic determined by A-scan rate, sampling density, the number of repeated B-scans, and motion-correction/averaging strategies. Prior work has shown that acquisition settings and image quality can systematically influence quantitative OCTA endpoints, emphasizing that speed-related differences may propagate into density-, topology-, and FAZ-related metrics if noise and artefacts are not rigorously controlled [12,13,25,26]. Reported acquisition durations for macular OCTA volumes vary across platforms and protocols [27], and scan-speed adjustments can measurably affect quantitative vascular metrics [28]. Therefore, acquisition time should be considered together with angiographic contrast and noise characteristics when OCTA is used for biomarker-driven studies or longitudinal monitoring [11].

From a clinical utility perspective, our findings suggest complementary strengths that should be aligned with the intended use case. For biomarker-driven macular quantification, especially when outcomes depend on DCP integrity and FAZ stability, high angiographic contrast and low background noise are critical, and rigorous quality control is required to avoid noise-driven inflation of density/topology metrics. For throughput-focused workflows and potentially wider-field imaging, ultrafast acquisition may reduce motion artefacts and facilitate imaging in patients with limited fixation; however, wide-field performance was not evaluated in the present study and should be assessed separately using dedicated wide-field protocols. Overall, these results support caution when comparing or pooling quantitative OCTA endpoints across platforms without harmonization of acquisition settings and analysis pipelines.

This study has several limitations. Firstly, only young, healthy eyes were included to maximize signal quality and to isolate device- and acquisition-related differences under standardized conditions. Therefore, generalizability to eyes with retinal pathology is limited, because media opacities, edema, hemorrhage, and ischemia-related flow deficits can attenuate OCTA signal, increase noise, and potentially alter device-specific susceptibility to artifacts as well as segmentation robustness. Secondly, manual FAZ initialization (seeding) was required in a subset of scans due to FAZ contamination by noise, which may introduce observer-dependent variability. Thirdly, projection artifacts were not explicitly quantified. In addition, our comparison was restricted to dense macular 3 × 3 mm scans; conclusions should not be extrapolated to wide-field protocols, which may exhibit different trade-offs between speed, artefacts, and quantitative robustness. Finally, multiple comparisons across several correlated OCTA endpoints were reported without formal multiplicity adjustment; thus, inference should be based primarily on effect sizes and the consistency of direction across metrics rather than isolated *p*-values. Future work should include diseased eyes, assess intra-device repeatability, and evaluate preprocessing approaches to reduce noise. Extension to larger fields and montage imaging will determine whether the observed differences persist beyond the central macula.

In conclusion, under a standardized high-density macular 3 × 3 mm protocol, BMizar achieved shorter acquisition times but exhibited higher background noise, which was associated with systematic shifts in density- and topology-related metrics and reduced FAZ robustness, particularly in the DCP. DREAM provided cleaner images with more stable FAZ delineation and higher vascular continuity, favoring biomarker-driven macular quantification.

From a practical standpoint, these results support application-specific recommendations. For biomarker-driven macular quantification under standardized conditions, particularly when robust DCP- and FAZ-dependent endpoints are required (e.g., studies focusing on macular ischemia markers or longitudinal microvascular monitoring), DREAM may be the preferable choice due to higher angiographic contrast, lower background noise, and greater FAZ stability in our protocol. In contrast, when acquisition speed and throughput are the dominant priorities (e.g., imaging in patients with limited fixation or high-throughput clinical workflows), BMizar may be advantageous, provided that stringent quality control is applied and quantitative endpoints are not pooled across platforms without harmonization of acquisition settings and analysis pipelines. Importantly, wide-field performance was not evaluated here and should be assessed separately using dedicated protocols.

## Figures and Tables

**Table 1:** Duration of image acquisition and corresponding interquartile range (IQR) for OCTA scans obtained with each system. Acquisition time is reported descriptively for the evaluated protocol.

	DREAM	BMizar
time (s)	9.93	5.36
IQR_time_ (s)	1.503	0.835

**Table 2:** Quantitative vascular metrics extracted from the en face images for superficial capillary plexus (SCP) as well as deep capillary plexus (DCP) across the OCTA devices. Values are reported as median (IQR), except for fractal dimension (FD) which is reported as mean (SD). *p*-values correspond to pairwise comparison and effect size is reported using Cliff’s delta (δ). Significant *p*-values in bold.

	DREAM (SCP)	BMizar (SCP)	DREAM (DCP)	BMizar (DCP)
VAD (%)	41.89	48.83	32.96	49.93
IQR_VAD_ (%)	2.23	1.63	2.11	1.06
FAZ (mm^2^)	0.2565	0.2370	0.2665	0.4240
IQR_FAZ_ (mm^2^)	0.1622	0.2060	0.1613	0.1795
TVL (mm)	174.2	188.1	223.6	206.2
IQR_TVL_ (mm)	12.1	7.7	14.6	6.9
Nodes	1547	1879	3144	2301
IQR_Nodes_	228	163	363	134
FD (mean)	1.980	1.973	1.980	1.970
SD_FD_	0.001979	0.004629	≈0	≈0
MVL (µm)	45	39		
IQR_MVL_ (µm)	3.25	1.00		
MVD (µm)			14	25
IQR_MVD_ (µm)			1	1
*p* (VAD)	**<0.0001**		**<0.0001**	
*p* (FAZ)	0.1413		**<0.0001**	
*p* (TVL)	**<0.0001**		**<0.0001**	
*p* (Nodes)	**<0.0001**		**<0.0001**	
*p* (FD)	**<0.0001**		**<0.0001**	
*p* (MVL)	**<0.0001**			
*p* (MVD)			**<0.0001**	
δ (VAD)	−1.00		−1.00	
δ (FAZ)	0.14		−0.92	
δ (TVL)	−0.84		0.84	
δ (Nodes)	−0.92		0.96	
δ (FD)	0.90		1.00	
δ (MVL)	1.00			
δ (MVD)			−1.00	

**Table 3:** Image quality parameters derived from the en face images of the two systems for superficial capillary plexus (SCP) and deep capillary plexus (DCP). Values are reported as median (IQR). *p*-values correspond to pairwise comparison and effect size is reported using Cliff’s delta (δ). Significant *p*-values in bold.

	DREAM (SCP)	BMizar (SCP)	DREAM (DCP)	BMizar (DCP)
FAZ-noise rate (%)	2.29	2.44	2.36	2.46
IQR_FAZ-noise rate_ (%)	0.215	0.050	0.205	0.050
CNR	16.650	0.820	7.560	0.685
IQR_CNR_	6.560	0.325	2.462	0.235
Noise-floor-SD (intensity units)	4.11	57.90	6.15	62.36
IQR_noise-floor-SD_ (Intensity units)	1.825	8.330	1.760	7.190
*p* (FAZ-noise rate)	**<0.0001**		**<0.0001**	
*p* (CNR)	**<0.0001**		**<0.0001**	
*p* (noise-floor-SD)	**<0.0001**		**<0.0001**	
δ (FAZ-noise rate)	−0.70		−0.76	
δ (CNR)	1.00		1.00	
δ (noise-floor-SD)	−1.00		−1.00	

## Data Availability

The data presented in this study are available on reasonable request from the corresponding author. The data are not publicly available due to privacy and ethical restrictions related to patient confidentiality.

## Abbreviations

The following abbreviations are used in this manuscript:

CNR	Contrast-to-noise ratio
DCP	Deep capillary plexus
FAZ	Foveal avascular zone
FD	Fractal dimension
IQR	Interquartile range
MVD	Mean vessel diameter
MVL	Median vessel length
OCT	Optical coherence tomography
OCTA	Optical coherence tomography angiography
OCTAVA	OCTA vascular analyser
ROI	Region of interest
SCP	Superficial capillary plexus
SD	Standard deviation
SS-OCTA	Swept-source optical coherence tomography angiography
TVL	Total vessel length
VAD	Vessel area density
δ	Cliff’s delta
